# MD/DPD Multiscale Framework for Predicting Morphology and Stresses of Red Blood Cells in Health and Disease

**DOI:** 10.1371/journal.pcbi.1005173

**Published:** 2016-10-28

**Authors:** Hung-Yu Chang, Xuejin Li, He Li, George Em Karniadakis

**Affiliations:** Division of Applied Mathematics, Brown University, Providence, Rhode Island, United States of America; University of California San Diego, UNITED STATES

## Abstract

Healthy red blood cells (RBCs) have remarkable deformability, squeezing through narrow capillaries as small as 3 microns in diameter without any damage. However, in many hematological disorders the spectrin network and lipid bilayer of diseased RBCs may be significantly altered, leading to impaired functionality including loss of deformability. We employ a two-component whole-cell multiscale model to quantify the biomechanical characteristics of the healthy and diseased RBCs, including *Plasmodium falciparum*-infected RBCs (*Pf*-RBCs) and defective RBCs in hereditary disorders, such as spherocytosis and elliptocytosis. In particular, we develop a *two-step multiscale framework* based on coarse-grained molecular dynamics (CGMD) and dissipative particle dynamics (DPD) to predict the static and dynamic responses of RBCs subject to tensile forcing, using experimental information only on the structural defects in the lipid bilayer, cytoskeleton, and their interaction. We first employ CGMD on a small RBC patch to compute the shear modulus, bending stiffness, and network parameters, which are subsequently used as input to a whole-cell DPD model to predict the RBC shape and corresponding stress field. For *Pf*-RBCs at trophozoite and schizont stages, the presence of cytoadherent knobs elevates the shear response in the lipid bilayer and stiffens the RBC membrane. For RBCs in spherocytosis and elliptocytosis, the bilayer-cytoskeleton interaction is weakened, resulting in substantial increase of the tensile stress in the lipid bilayer. Furthermore, we investigate the transient behavior of stretching deformation and shape relaxation of the normal and defective RBCs. Different from the normal RBCs possessing high elasticity, our simulations reveal that the defective RBCs respond irreversibly, *i.e.*, they lose their ability to recover the normal biconcave shape in successive loading cycles of stretching and relaxation. Our findings provide fundamental insights into the microstructure and biomechanics of RBCs, and demonstrate that the *two-step multiscale framework* presented here can be used effectively for *in silico* studies of hematological disorders based on first principles and patient-specific experimental input at the protein level.

## Author Summary

Red blood cells (RBCs) and their mechanical properties play a crucial role in the dynamic and rheological behavior of blood in normal and disease states. However, the precise determination of RBC membrane properties is hard to be achieved experimentally. To this end, accurate numerical modeling can be used to provide valuable information for quantifying the biomechanical properties of RBCs. In this paper, we have developed and validated a *two-step multiscale framework* for RBC modeling, by performing molecular dynamics simulations to compute the shear modulus, bending stiffness and network parameters of a small RBC patch, which we then use as input to dissipative particle dynamics simulations to predict the stress field and morphology of defective RBCs, including *Plasmodium falciparum*-infected RBCs as well as RBCs in hereditary spherocytosis and elliptocytosis.

## Introduction

Blood is a biological fluid that delivers nutrients and oxygen to living cells and removes their waste products. Due to its particulate nature, blood is considered as a complex non-Newtonian fluid exhibiting intriguing dynamic and rheological behavior depending mainly on flow rate, vessel geometry, and volume fraction of suspending particles, especially red blood cells (RBCs).

The deformability of a RBC is determined by the geometry, elasticity, and viscosity of its membrane [1, 2]. A healthy RBC (H-RBC) has a biconcave shape when not subject to any external stress and is approximately 8.0 *μm* in diameter and 2.0 *μm* in thickness. The membrane of a RBC consists of a lipid bilayer contributing to the bending resistance, an attached spectrin network (cytoskeleton) responsible for the shear stiffness, and transmembrane proteins such as band-3 and glycophorin C, bridging the connections between lipid and spectrin domains. Experimental and numerical observations of RBC behavior in flow mimicking the microcirculation reveal dramatic deformations and rich dynamics. The extreme deformability allows RBCs to squeeze through narrow capillaries in microcirculation without any damage. However, this feature of RBCs can be critically affected by parasitic conditions such as malaria [2, 3], or genetic factors, e.g., in sickle cell disease (SCD) [4, 5], hereditary spherocytosis (HS) [6], and hereditary elliptocytosis (HE) [7]. For many diseases involving RBCs, it is known that the membrane damage and interactions associated with the lipid bilayer and the cytoskeleton including elastic strength, viscous friction, and integral protein linkages strongly influence the biomechanics of RBCs [8]. For example, RBCs infected with *Plasmodium falciparum* (*Pf*-RBCs) become progressively less deformable and more spherical during the intraerythrocytic cycle. Remarkable nanoscale protrusions (knobs) have recently been identified causing significant stiffening effects on the cell membrane [9, 10]. Knobs mainly composed of two exported parasite proteins, knob-associated histidine-rich protein (KAHRP) and parasite-derived erythrocyte membrane protein (PfEMP1), deposit at the cytoplasmic face of RBC membrane and form vertical links to spectrin network. Consequently, both the lipid bilayer and the spectrin network are stiffened by the knobs as it is implied by the notable enhancement in shear resistance. In addition to the presence of knobs, actin mining introduced by the parasite invasion may result in an enhanced spectrin network at the trophozoite stage or in a deficient spectrin network at the schizont stage [10, 11].

SCD is an inherited blood disorder exhibiting heterogeneous cell morphology and abnormal rheology, due to the polymerization of sickle hemoglobin at high enough concentrations, forming long fibers that distort the RBC shape and dramatically alter their biomechanical properties [4, 12]. HS RBC is usually caused by the defects in anchoring proteins involved in vertical interactions between lipid bilayer and spectrin network, whereas HE RBC is a result of defects in spectrin filaments related to lateral interactions in the spectrin network [13]. Protein mutations associated with membrane defects subsequently lead to aberrant cell shape and impaired deformability. Thus, quantifying the deformability of RBCs could play a key role in understanding RBC related diseases.

Recent advances in computational modeling and simulation enables us to tackle a broad range of dynamics and rheology problems associated with RBCs [14–16]. Several computational models at the whole-cell level, including spectrin-level and multiscale RBC models [17–23], have been developed and employed to quantify the biomechanical properties and dynamic behavior of RBCs in malaria and other hematological diseases [24–29]. Examples include dynamic cell deformability for various stages of the *Pf*-RBCs [24–26, 30–33] and cell morphological sickling [34–36] and vaso-occlusion phenomena in SCD [27, 37]. In these existing models, the membrane is usually considered as a single shell with effective properties that represent the combined effects of lipid bilayer and spectrin network. Under normal conditions, the cytoskeleton is attached to the lipid bilayer from the cytoplasmic side. However, under certain conditions, such as RBCs in SCD and other hereditary disorders, the cytoskeleton may become dissociated from the lipid bilayer [12, 13]. Also, in malaria disease, the *Pf*-RBC undergoes irreversible structural modifications with the deposition of knob structures on the membrane surface and the actin remodeling in the skeletal network [38, 39]. The biomechanical properties associated with the bilayer-cytoskeleton interactions strongly influence biorheology, cell function and the onset and progression of RBC diseases. However, the one-component whole-cell models cannot facilitate detailed whole-cell exploration of diverse biophysical and biomechanical problems involving RBCs, such as the bilayer loss in HS, the bilayer-cytoskeleton uncoupling in SCD and the aforementioned multiple stiffening effects of knob structures in *Pf*-RBCs. For these reasons, there is a compelling need to develop a more realistic RBC representation, e.g., to endow the spectrin-based RBC models with more accurate structure, hence considering separately the lipid bilayer and cytoskeleton but also include the transmembrane proteins.

Recent efforts have been directed towards this approach. For example, a two-component composite model of RBC membrane with explicit descriptions of lipid bilayer, cytoskeleton, and transmembrane proteins has been developed and implemented using coarse-grained molecular dynamics (CGMD) [10, 41, 42]. This CGMD membrane model has been successfully applied to study the membrane-related problems in RBCs such as protein diffusion and vesiculation in defective RBC membrane [43, 44], and the stiffening effects of knobs on *Pf*-RBCs [10]. Although changes on the biomechanics of RBC membrane, including bending rigidity and shear modulus, in certain diseases can be evaluated by modeling a small piece of cell membrane with the two-component composite model, the whole-cell characteristics strongly related to RBC biomechanics and biorheology are not efficiently depicted by modeling only a portion of the RBC membrane. Recently, a two-component whole-cell model has been developed and implemented using dissipative particle dynamics (DPD) [45, 46]. The DPD RBC model also accounts separately for the lipid bilayer and cytoskeleton, but it includes implicitly the transmembrane proteins, Thus, the DPD model is computationally more efficient than CGMD model for RBC modeling at the whole-cell level, which has been applied to investigate RBC response and dynamics in flow. However, the lack of the molecular details in this two-component whole-cell model may limit its predictive capacity in identifying the key factors that cause the reorganization of the RBC membrane. An effective way to address this problem is to incorporate only the necessary molecular information from a molecular-detailed composite membrane model into a more coarse-grained whole-cell model.

**Fig 1:**
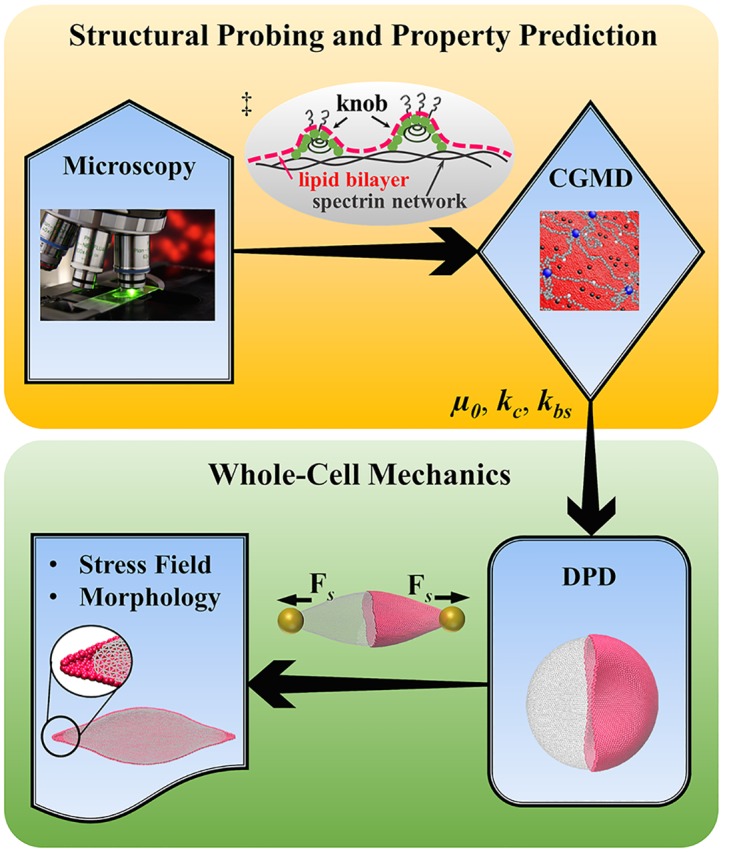
Two-step multiscale framework for RBC modeling. The experimental information about the structural defects of the lipid bilayler, the cytoskeleton and their coupling via the transmembrane proteins is collected and considered as input to two-component composite CGMD model. The CGMD is then employed on a small RBC patch to compute the shear modulus (*μ*_0_), bending stiffness (*k*_*c*_), and network parameters (*k*_*bs*_), which are subsequently used as input to a whole-cell DPD model to predict the RBC shape and corresponding stress field. ‡ A schematic diagram of nanoscale knob on the membrane surface of a *Pf*-RBC.

In this work, we develop a *two-step multiscale framework* by employing the two-component models; see Fig 1. The only experimental input required is information about the structural defects of the lipid bilayer, the cytoskeleton, and their coupling via the transmembrane proteins. This information can be obtained by Scanning Electron Microscopy (SEM) [47] or Transmission Electron Microscopy (TEM) [40]. We then apply this framework to study the biomechanical characteristics of the healthy and diseased RBCs under static and dynamic tensile forcing. Specifically, we probe the alterations in cell deformability from the development of knob structures and remodeling of the spectrin network of *Pf*-RBCs. We also assess the implication of dynamic deformation and recovery response of defective RBCs from the bilayer-cytoskeleton dissociation in hereditary disorders such as HS and HE.

The rest of the paper is organized as follows. In Section 2, we briefly describe the RBC model and simulation method. In Section 3, we present and discuss our numerical results. Finally, in Section 4, we summarize the findings and present the conclusion. We also provide supplementary material in order to better explain the simulation results.

## Methods and Models

In the first step of the two-step process, we compute the shear modulus, bending stiffness, and network parameters by employing CGMD on a small RBC patch. In the second step, by passing the aforementioned parameters as input to a whole-cell DPD model, we simulate the stretching deformation, stress field, and shape relaxation of the normal and diseased RBCs subject to tensile forcing. For completeness, the simulation methods and models are briefly reviewed below, whereas details on the construction of RBC models are available elsewhere [10, 42, 45, 46].

### Two-component CGMD model of RBC membrane

**Fig 2:**
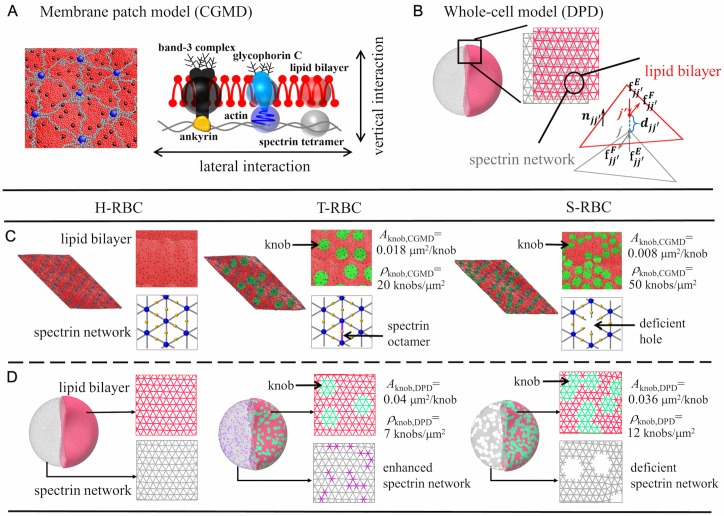
Schematic representation of the two-component composite CGMD model (A & C) and whole-cell DPD model (B & D) of H-RBCs and *Pf*-RBCs. For the composite CGMD model, the red, blue, and grey particles represent clusters of lipid molecules, actin junctions, and actin filaments of cytoskeleton, respectively; the black and yellow particles signify band-3 complexes, of which one third (yellow ones) are connected to the spectrin network; the green patches represent the rigid knobs in the *Pf*-RBC membrane; the purple particles refer to the spectrin octamers. For the whole-cell DPD model, the lipid bilayer and cytoskeleton are rendered in red and grey triangular networks, respectively. Only half of the triangular network of the lipid bilayer is shown for clarity. The rigid knobs in lipid bilayer of the *Pf*-RBC is rendered in green, while the enhanced spectrin network of T-RBC and deficient spectrin network of S-RBC are highlighted in purple bonds and visible holes in the triangular network of the cytoskeleton. The knob density in the whole-cell DPD model is set to be lower than that in the composite CGMD model due to different levels of coarse-graining applied to these two-component models. In the whole-cell DPD model, the average size of a knob (*A*_knob,DPD_) is around 0.04 *μ*m^2^ and 0.036 *μ*m^2^ for T-RBC and S-RBC, respectively, which is around 2–5 times bigger than that (*A*_knob,CGMD_) used in the composite CGMD model. Thus, *ρ*_knob,DPD_ ≈ (0.2–0.5)*ρ*_knob,CGMD_.

In the two-component CGMD model of a normal RBC membrane, the lipid bilayer and cytoskeleton as well as the transmembrane proteins are explicitly represented by CGMD particles [41]. Specifically, three types of CG particles are introduced to represent the lipid bilayer of the RBC membrane (Fig 2A and 2C). The red particles represent clusters of lipid molecules with a diameter of 5 nm, which is approximately equal to the thickness of the lipid bilayer; the black-color particles signify band-3 complexes; the light blue particles immersed in the lipid bilayer are glycophorin C. The volume of one black particle is similar to the excluded volume of the membrane domain of a band-3 protein. However, when band-3 proteins interact with the cytoskeleton, the effect of the cytoplasmic domain has to be taken into account and thus the effective radius is ≈ 12.5 nm. One third of band-3 particles, which are connected to the spectrin network, are depicted as yellow particles (Fig 2C).

The spectrin tetramer is modeled by a chain of 39 beads (grey particles) connected by spring bonds [48]. The corresponding potential has the form,

$$V_{cy}^{s-s}=\frac{1}{2}k_{0}{\left(r-r_{eq}^{s-s}\right)}^{2}$$ (1)

where *k*_0_ and $r_{eq}^{s-s}$ are the spring constant and equilibrium distance between two spectrin particles, respectively. Spectrin particles that are not connected by the spring potential interact with each other via the repulsive term of the Lennard-Jones potential as follows

$$V_{rep}\left(r_{ij}\right)=\left\{\begin{array}{cc} 4\epsilon \left[{\left(\frac{\sigma_{ij}}{r_{ij}}\right)}^{12}-{\left(\frac{\sigma_{ij}}{r_{ij}}\right)}^{6}\right]+\epsilon & \;r_{ij}<r_{eq}^{s-s}\\ 0 & \;r_{ij}\geq r_{eq}^{s-s}\; \end{array}\right.$$ (2)

where *ϵ* is the energy unit and *σ*_*ij*_ is the length unit, and *r*_*ij*_ is the distance between spectrin particles.

To couple the lipid bilayer and spectrin network, actin junctional complexes (blue particles) are connected to the glycophorin C and the middle beads of the spectrin network are bonded to the band-3 complexes (black particles) which are specifically rendered in yellow particles, as shown in Fig 2C. These bonds are modeled as harmonic springs, given by

$$V_{cy}^{a-s}=\frac{1}{2}k_{0}{\left(r-r_{eq}^{a-s}\right)}^{2}$$ (3)

where $r_{eq}^{a-s}$ = 10 nm is the equilibrium distance between an actin and a spectrin particle. For a detailed description of the configuration of the cell membrane and the employed potentials, we refer to Ref. [42].

#### *Pf*-RBC membrane

In order to represent the knobs observed in *Pf*-RBCs, we introduce a new type of CG lipid particle (rendered in green in Fig 2C). Since the knob regions are more rigid than the normal lipid bilayer, the association energy between green particles is twice as much as the association energy between the red particles and between the red and green particles. Based on the analysis of knob structure of *Pf*-RBCs by SEM [47], the knobs have radius around 55–80 nm and density around 10–35 knobs/*μ*m^2^ at the trophozoite stage; the knob size is decreased to 35–50 nm while the density is increased to 45–70 knobs/*μ*m^2^ at the schizont stage. In this study, we follow the previous CGMD simulations [10], where the average knob radius is decreased from 75 to 50 nm, and the knob density (*ρ*_knob,CGMD_) is increased from 20 to 50 knobs/*μ*m^2^ through the parasite development from trophozoite to schizont stages (see the T-RBC and S-RBC in Fig 2C). The center of each knob is placed on the top of actin junction. Each knob could form vertical links when interacting with the actin junctions, the 4-th and the 19-th particles of the spectrin filaments where the ankyrins are located. In addition to the formation of knobs, spectrin remodeling due to the actin mining generates two likely modified spectrin networks. One is an enhanced spectrin network with partial spectrin tetramers replaced by spectrin octamers, and the other one is a deficient spectrin network with noticeable holes formed in the spectrin network caused by the loss of actin oligomers [10, 11]. For T-RBC, we replace 20% spectrin tetramers (grey particles) by spectrin octamers (purple particles); for S-RBC, we remove 14% actin junctions (blue particles) from the spectrin network resulting in the formation of deficient holes (see Fig 2C).

**Fig 3:**
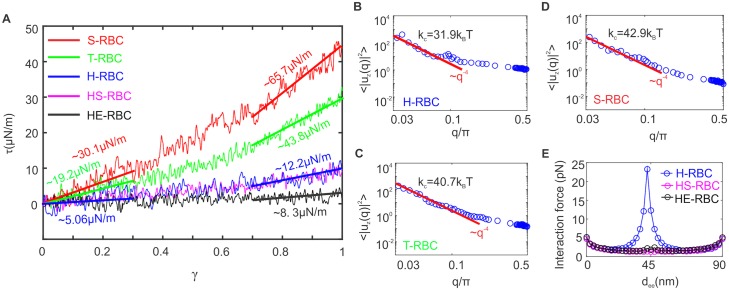
Biomechanical properties obtained from two-component composite CGMD model. (**A**) Shear response of the healthy and diseased RBC membranes. (**B**)-(**D**) show the vertical displacement fluctuation spectra of healthy and diseased RBC membranes as a function of wave number *q*. (**E**) Measured bilayer-cytoskeleton interaction force at different end-to-end distance between two actin junctions, *d*_*ee*_, for healthy and defective RBCs.

In order to compute the shear modulus of the RBC membrane from CGMD simulations, the cell membrane is sheared up to a shear strain (*γ*) of “1”. The shear response of the RBC membrane is illustrated in Fig 3A. For the H-RBC membrane, the shear modulus of the membrane is ∼ 5 *μ*N/m at small deformation while it is increased to ∼ 12 *μ*N/m at large deformation, which is consistent with the experimentally measured values of 4–12 *μ*N/m [49]. For T-RBC, the multiple stiffening effect of knobs enhances the shear modulus of the RBC membrane to ∼ 19 and ∼ 44 *μ*N/m at low and high shear strains, respectively (green line in Fig 3A). For S-RBC, the shear modulus is further increased to ∼ 30 and ∼ 66 *μ*N/m at low and high shear strains, respectively (red line in Fig 3A). These results demonstrate that the development of knob structures and their density play significant roles in elevating the shear modulus of *Pf*-RBCs. In addition, these values are consistent with the measured shear moduli lie between 20 *μ*N/m and 50 *μ*N/m for T-RBC and between 40 *μ*N/m and 90 *μ*N/m for S-RBC [2, 8, 50]. For comparison, we summarized all of the data in Table 1.

**Table 1:** Shear moduli of RBCs at different pathological conditions.

RBC state	Shear modulus (*μ*N/m)			
	Simulation		Experiment	Reference
	small *γ*	high *γ*		
**H-RBC**	∼ 5	∼ 12	4–12	[8, 49]
**T-RBC**	∼ 19	∼ 44	20–50	[2, 8, 50]
**S-RBC**	∼ 30	∼ 66	40–90	[2, 8, 50]
**HS-RBC**	∼ 5	∼ 12	∼ 7.6	[56]
**HE-RBC**	∼ 5	∼ 8	∼ 5.1	[56]

The bending stiffness of the RBC membrane is obtained by applying the equipartition theorem on the Helfrich free energy in the Monge representation [51–54], which is represented by

$$\left\langle |u_{z}\left(q\right){|}^{2}\right\rangle =\frac{k_{B}T}{l^{2}\left(\sigma q^{2}+k_{c}q^{4}\right)},$$ (4)

where *l* is equal to the CGMD particle size, *σ* is the surface tension, *k*_*c*_ is the bending modulus, and *u*_*z*_(*q*) is the discrete Fourier transform of the out-of-plane displacement $\tilde{u}\left(r\right)$ of the membrane. To compute the bending modulus, *k*_*c*_, we calculate |*u*_*z*_(*q*)|^2^ from the membrane thermal fluctuation data (blue circles in Fig 3B–3D) and fit them to Eq (4) at a stress-free state, *σ* = 0 (*q*^−4^ in Fig 3B–3D). We obtain *k*_*c*_ = 31.9, 40.7 and 42.9 *k*_B_ *T* for the membranes of H-RBC, T-RBC, and S-RBC, respectively.

#### Defective RBC membrane in hereditary spherocytosis and elliptocytosis

For HS-RBC, its membrane loses integrity due to the defects in transmembrane proteins that connect the spectrin network to the lipid bilayer [6, 55]. Therefore, we remove the connections between the spectrin filaments and immobile band-3 proteins in the RBC membrane to represent a defective membrane in HS. On the other hand, HE-RBC has an impaired membrane mechanical stability due to the defects in membrane skeletal proteins [13, 55]. In this work, we model such defects by reducing the cytoskeleton connectivity. Specifically, we have studied the shear responses of RBC membrane with different cytoskeleton connectivity, see S1 Fig, which shows that the shear modulus decreases as cytoskeleton connectivity is reduced. According to the experimental studies by Waugh *et al.* [56], the shear modulus of HE-RBC (∼ 5.1 *μ*N/m) is somewhat lower compared to that of H-RBC (∼ 6.7 *μ*N/m). Based on their results, we chose a moderate reduction in the spectrin connections (*e.g.* 60% cytoskeleton connectivity), to qualitatively represent the defects in HE. As shown in Fig 3A and Table 1, the estimated shear modulus of HE-RBC is a little smaller than that of HS-RBC, which is also consistent with the experimental observations.

For defective RBCs in HS and HE, the interactions between the cytoskeleton and lipid bilayer could be significantly weakened due to the protein abnormalities. We then employ CGMD on a small RBC patch to estimate the average bilayer-cytoskeleton interaction strength, see Fig 3E. For normal RBC membrane, the spectrin filaments are anchored to the lipid bilayer via band-3 proteins at the spectrin-ankyrin binding sites, placed in the middle of two actin junctions. The simulation results in Fig 3E show that the strength of bilayer–cytoskeleton interaction is on the order of ∼ 25 pN, when we choose the value located at the half of the end-to-end distance between two actin junctions, *i.e.*, *d*_*ee*_ ∼ 45 nm. This value is of the same order of magnitude as those measured or estimated in previous studies, which are ranged from 10 to 60 pN [45, 57, 58]. By contrast, the interaction strength for HS-RBCs and HE-RBCs at *d*_*ee*_ = 45 nm drops significantly to ∼ 2.5 pN, which is an order of magnitude lower than that for H-RBCs.

### Two-component whole-cell DPD model

In the two-component whole-cell model, the cell membrane is modeled by two distinct components, *i.e.*, the lipid bilayer and the spectrin network (Fig 2B). Specifically, through the DPD approach, each component is constructed by a 2D triangulated network on a membrane surface that is characterized by a set of points with Cartesian coordinates **x**_*i*_, *i* ∈ 1 ⋯ *N*_*v*_ which are vertices of the 2D triangulated network. Different from the one-component whole-cell model [20, 21], the lipid bilayer of the two-component whole-cell model has no shear stiffness at healthy state but only bending stiffness and a very large local area stiffness, whereas the cytoskeleton has no bending stiffness but possesses a finite shear stiffness.

The whole-cell DPD model takes into account the elastic energy, bending energy, bilayer-cytoskeleton interaction energy, and constraints of fixed surface area and enclosed volume, hence

$$V\left(x_{i}\right)=V_{s}+V_{b}+V_{a}+V_{v}+V_{int}$$ (5)

where *V*_*s*_ is the elastic energy that mimics the elastic spectrin network, given by

$$V_{s}=\sum_{j\in 1...N_{s}}\left[\frac{k_{B}Tl_{m}\left(3x_{j}^{2}-2x_{j}^{3}\right)}{4p(1-x_{j})}+\frac{k_{p}}{\left(n-1\right)l_{j}^{n-1}}\right],$$ (6)

where *l*_*j*_ is the length of the spring *j*, *l*_*m*_ is the maximum spring extension, *x*_*j*_ = *l*_*j*_/*l*_*m*_, *p* is the persistence length, *k*_B_*T* is the energy unit, *k*_*p*_ is the spring constant, and *n* is a specified exponent. The shear modulus of the RBC membrane, *μ*_0_, is determined by

$$\mu_{0}=\frac{\sqrt{3}k_{B}T}{4pl_{m}x_{0}}\left(\frac{x_{0}}{2{\left(1-x_{0}\right)}^{3}}-\frac{1}{4{\left(1-x_{0}\right)}^{2}}+\frac{1}{4}\right)+\frac{\sqrt{3}k_{p}\left(n+1\right)}{4l_{0}^{n+1}},$$ (7)

where *l*_0_ is the equilibrium spring length and *x*_0_ = *l*_0_/*l*_*m*_. The bending resistance of the RBC membrane is modeled by

$$V_{b}=\sum_{j\in 1...N_{s}}k_{b}\left[1-cos(\theta_{j}-\theta_{0})\right],$$ (8)

where *k*_*b*_ is the bending constant, *θ*_*j*_ is the instantaneous angle between two adjacent triangles having the common edge *j*, and *θ*_0_ is the spontaneous angle.

Constraints on the area and volume conservation of RBC are imposed to mimic the area-preserving lipid bilayer and the incompressible interior fluid. The corresponding energy is given by

$$V_{a+v}=\sum_{j\in 1...N_{t}}\frac{k_{d}{\left(A_{j}-A_{0}\right)}^{2}}{2A_{0}}+\frac{k_{a}{\left(A_{\text{cell}}-A_{\text{cell},0}^{\text{tot}}\right)}^{2}}{2A_{\text{cell},0}^{\text{tot}}}+\frac{k_{v}{\left(V_{\text{cell}}-V_{\text{cell},0}^{\text{tot}}\right)}^{2}}{2V_{\text{cell},0}^{\text{tot}}},$$ (9)

where *N*_*t*_ is the number of triangles in the membrane network, *A*_0_ is the triangle area, and *k*_*d*_, *k*_*a*_ and *k*_*v*_ are the local area, global area and volume constraint coefficients, respectively. The terms $A_{\text{cell},0}^{\text{tot}}$ and $V_{\text{cell},0}^{\text{tot}}$ represent the specified total area and volume, respectively.

The bilayer-cytoskeleton interaction potential, *V*_*int*_, is expressed as a summation of harmonic potentials given by

$$V_{int}=\sum_{j,j^{\prime }\in 1...N_{bs}}\frac{k_{bs}{\left(d_{jj^{\prime }}-d_{jj^{\prime },0}\right)}^{2}}{2},$$ (10)

where *k*_*bs*_ and *N*_*bs*_ are the spring constant and the number of bond connections between the lipid bilayer and the cytoskeleton, respectively. *d*_*jj*′_ is the distance between the vertex *j* of the cytoskeleton and the corresponding projection point *j*′ on the lipid bilayer, with the corresponding unit vector **n**_*jj*′_; *d*_*jj*′,0_ is the initial distance between the vertex *j* and the point *j*′, which is set to zero in the current simulations. Physical view of the local bilayer-cytoskeleton interactions include the major connections via band-3 complex and ankyrin, as well as the secondary connections via glycophorin C and actin junctions (Fig 2A), here we consider them together as an effective bilayer-cytoskeleton interaction and model it as a normal elastic force, $f_{jj^{\prime }}^{E}$, and a tangential friction force, $f_{jj^{\prime }}^{F}$ (Fig 2B). The corresponding elastic force on the vertex *j* of the cytoskeleton is given by

$$f_{jj^{\prime }}^{E}=\left\{\begin{array}{cc} k_{bs}\left(d_{jj^{\prime }}-d_{jj^{\prime },0}\right)n_{jj^{\prime }} & \;d_{jj^{\prime }}<d_{c}\\ 0 & \;d_{jj^{\prime }}\geq d_{c} \end{array}\right.$$ (11)

where *d*_*c*_ ≈ 0.2 *μm* is the cutoff distance. The tangential friction force between the two components is represented by

$$f_{jj^{\prime }}^{F}=-f_{bs}\left[v_{jj^{\prime }}-\left(v_{jj^{\prime }}\cdot n_{jj^{\prime }}\right)n_{jj^{\prime }}\right],$$ (12)

where *f*_*bs*_ is the tangential friction coefficient and **v**_*jj*′_ is the difference between the two velocities.

The RBC membrane interacts with fluid particles through DPD conservative forces, which are defined as

$$F_{ij}^{C}=a_{ij}{\left(1-r_{ij}/r_{c}\right)}^{s}n_{ij},$$ (13)

where **r**_*ij*_ = **r**_*i*_ − **r**_*j*_, *r*_*ij*_ = |**r**_*ij*_|, **n**_*ij*_ = **r**_*ij*_/*r*_*ij*_, *a*_*ij*_ is the maximum repulsion between particles *i* and *j*, *r*_*c*_ is the cutoff distance, *s* = 1 is the most widely adopted for the classical DPD method. However, other choices (e.g., *s* = 0.25) for the envelopes have also been used. Detailed description of these interations can be found in Ref. [21]. In addition, in combination with the total energy *V*(**x**_*i*_) expressed in Eq 5, we are able to derive the total force acting on particle *i* of the RBC membrane,

$$F_{i}=-\frac{\partial V(x_{i})}{\partial x_{i}}+\sum_{j\neq i}F_{ij}^{C}.$$ (14)

The RBC model is multiscale, as the RBC can be represented on the spectrin level (N_*v*_^DPD,S^ = 23,867), where each spring in the network corresponds to a single spectrin tetramer with the equilibrium distance between two neighboring actin connections of ∼ 75 nm [20, 59], equal to the average length of one edge of triangular mesh of RBC membrane in the CGMD model. In such case, both CGMD and DPD simulate the RBC membrane at the spectrin level [21, 44]. Thus, the size and density of nanoscale knobs of *Pf*-RBCs applied in both CGMD and DPD models can be directly derived from measured data in experiments. In addition, the RBC membrane properties, such as shear modulus *μ*_0_ and bending stiffness *k*_*c*_ can be passed directly from CGMD to DPD,

$$l_{0}^{\text{DPD},S}=l_{0}^{\text{CGMD},S},\;\mu_{0}^{\text{DPD},S}=\mu_{0}^{\text{CGMD},S},\;k_{c}^{\text{DPD},S}=k_{c}^{\text{CGMD},S}.$$ (15)

On the other hand, for more efficient computation, the RBC network can also be coarse-grained by using a smaller number of vertices. The equilibrium spring length of the coarse-grained RBC model is then estimated as:

$$l_{0}^{\text{DPD},C}=l_{0}^{\text{DPD},S}\left(\sqrt{\frac{{N_{v}}^{\text{DPD},S}-2}{{N_{v}}^{\text{DPD},C}-2}}\right).$$ (16)

Using a similar geometric argument, the spontaneous angle is adjusted as,

$$\theta_{0}^{\text{DPD},C}=\theta_{0}^{\text{DPD},S}\left(l_{0}^{\text{DPD},S}/l_{0}^{\text{DPD},C}\right).$$ (17)

In addition, the property parameters of the RBC membrane can be estimated as,

$$\mu_{0}^{\text{DPD},C}=\mu_{0}^{\text{DPD},S},\;k_{c}^{\text{DPD},C}=k_{c}^{\text{DPD},S}.$$ (18)

In this study, we adopt a whole-cell DPD model with *N_v_*^DPD,C^ = 9128 in order to simulate the stretching behavior of the whole RBC more effectively. We model an H-RBC using the whole-cell DPD model with the following properties: $A_{\text{cell},0}^{\text{tot}}$ = 134 *μm*^2^, $V_{\text{cell},0}^{\text{tot}}$ = 94 *μm*^3^. Based on the CGMD and previous one-component whole-cell simulations of [21], we choose *μ*_0_ = 4.73 *μ*N/m for H-RBC. Regarding the elastic contribution to the interaction energy, we use the value of the bending modulus derived directly from CGMD simulations, *i.e.*, *k*_*c*_ = 31.9 *k*_B_*T* for H-RBC, which is approximately 1.3 ×10^−19^ J. The corresponding bending constant is set to $k_{b}=\frac{2}{\sqrt{3}}k_{c}$ = 36.8 *k*_B_*T*. It has been shown that the bending resistance contributes little to the cell deformation in the stretching test in previous studies [23, 26, 33], so we keep *k*_*b*_ constant in all simulation cases.

The simulations are performed using a modified version of the atomistic code LAMMPS. The time integration of the motion equations is computed through a modified velocity-Verlet algorithm [60] with *λ* = 0.50 and time step Δ*t* = 1.0 × 10^−5^ *τ* ≈ 0.23 *μs*. It takes 2.0 ×10^6^ time steps for a typical simulation performed in this work.

#### *Pf*-RBCs

Similar to the two-component CGMD model for *Pf*-RBC membrane, the knobs are modeled as rigid patches in the lipid domain, which are randomly chosen depending on the knob density (see the green patches in Fig 2D). However, different from the full resolution RBC level in CGMD models having an average spectrin length ∼ 75 nm, the equilibrium spring length ($l_{0}^{\text{DPD},C}$) in our whole-cell DPD model is ∼ 119 nm (based on Eq 16) at a coarse-grained level *N_v_*^DPD,C^ = 9128. Thus, a knob size is around 0.04 *μ*m^2^ at the trophozoite stage, which is 2.5 times bigger than that observed in experiments [47]. To keep the ratio of the total knobby area to RBC membrane area at the same value, *ρ*_knob,DPD_ ≈ 0.4 *ρ*_knob,CGMD_ at the trophozoite stage is defined, *i.e.* *ρ*_knob,DPD_ ≈ 8 knobs/*μ*m^2^ if *ρ*_knob,CGMD_ ≈ 20 knobs/*μ*m^2^. Likewise, a smaller knob size 0.036 *μ*m^2^ at the schizont stage due to shrinkage of cell volume, is around 4.5 times larger than that of a knob with radius 50 nm in experiments [47]. An approximation of knob density at the schizont stage is denoted as, *ρ*_knob,DPD_ ≈ 0.22 *ρ*_knob,CGMD_, *i.e.* *ρ*_knob,DPD_ ≈ 11 knobs/*μ*m^2^ if *ρ*_knob,CGMD_ ≈ 50 knobs/*μ*m^2^. In this work, we have investigated the influence of the knob density on RBC deformability. The *ρ*_knob,DPD_ is changed from 4 to 9 knobs/*μ*m^2^ for T-RBC and from 10 to 18 knobs/*μ*m^2^ for S-RBC, which are also in reasonable ranges at each *Pf*-infected stage [47]. Moreover, the remodeling of the spectrin network in the *Pf*-RBC is achieved by modifying the elastic bonds between vertices in the cytoskeleton. Specifically, for T-RBC, 20% of elastic bonds in the cytoskeleton are twice stiffer than the normal ones to represent the replacement of spectrin tetramers by octamers. On the other hand, to mimic the 20% reduction in the specrtrin abundance from normal RBC to S-RBC [11], we have modified our whole-cell model by removing 14% and 22% vertices in the spectrin network. S1 Table shows the elongation index (EI) of S-RBC with different knob density and defective spectrin network at stretching force 110 pN. The elongation index (EI) is estimated based on the axial (*D*_A_) and transverse (*D*_T_) diameters of stretched RBCs,

$$\text{EI}=\frac{D_{A}-D_{T}}{D_{A}+D_{T}}.$$ (19)

It is commonly used to determine the deformability of RBC, and the larger the value, the more deformable the RBC is [61]. In general, we find that the EI value decreases with the increase in the knob density, but increases when the spectrin network loses more connections. However, the difference in the EI values between 14% and 22% deficiency of spectrin network becomes negligible when the knob density is relatively high ∼ 18 knobs/*μ*m^2^. In all simulations on S-RBC that follow we used 14% deficiency of spectrin network.

The parameters employed for modeling *Pf*-RBCs are the same as those for H-RBCs, except for the following parameters: shear modulus *μ*_0_, RBC surface area $A_{\text{cell},0}^{\text{tot}}$ and volume $V_{\text{cell},0}^{\text{tot}}$. It is widely acceptable that the invasion of *Plasmodium falciparum* into RBC brings different influences on lipid bilayer and cytoskeleton components [40, 62, 63]. However, current experimental studies have not been able to determine the molecular details, and thus, it is difficult to separate the effects of these two different components. Computational modeling offers an alternative method in investigating the underlying mechanisms, for example, Zhang *et al.* recently revealed the appreciable shear stress emerging at knobby regions in the lipid bilayer by CGMD simulations [10]. Similarly, in our CGMD simulations, shear responses of RBC membrane at the trophozoite and schizont stages, are reflected not only on the modified spectrin network but also on the stiffened lipid bilayer (see S2 Fig). In addition, following the concept of effective shear modulus of RBC membrane, comprising the lipid bilayer and spectrin network in previous experimental studies [2], we define an effective shear modulus (*μ*_*eff*_) of RBC membrane having the shear contributions from the reorganized lipid bilayer and spectrin network. According to our CGMD results, we have *μ*_*eff*_ ≈ 32 *μ*N/m in T-RBC, *i.e.* the mean value of blue curve in S2 Fig or the green curve in Fig 3A, and *μ*_*eff*_ ≈ 48 *μ*N/m in S-RBC, which are regarded as the inputs to our whole-cell DPD model. When the knob density increases, the stiffened lipid bilayer would contribute more to the effective shear modulus. Moreover, we consider the cell size change of *Pf*-RBCs at different stages into the simulations, *i.e.*, we set $A_{\text{T},0}^{\text{tot}}$ = 117 *μm*^2^ and $V_{\text{T},0}^{\text{tot}}$ = 77 *μm*^3^ for T-RBC and $A_{\text{S},0}^{\text{tot}}$ = 105 *μm*^2^ and $V_{\text{S},0}^{\text{tot}}$ = 65 *μm*^3^ for S-RBC.

#### Defective RBCs in hereditary spherocytosis and elliptocytosis

The membrane of defective RBC in HS and HE is modeled similarly as the one of normal RBC, except the interaction between the lipid bilayer and cytoskeleton. As a result, parameters specified for modeling defective RBC remain the same as those for H-RBC, except for the strength of the bilayer–cytoskeleton interactions. Following Peng *et al.* [45], we set the default values of *k*_*bs*_ = 46 pN/*μ*m and *f*_*bs*_ = 0.194 pN/*μ*m s for H-RBCs in the current study. As suggested from the CGMD simulations (Fig 3E), the interaction strength between the lipid bilayer and cytoskeleton of RBCs in HS and HE is decreased at least by one order of magnitude, so we change *k*_*bs*_ in the range from 46 pN/*μ*m to 0.46 pN/*μ*m, and *f*_*bs*_ from 0.194 pN/*μ*m s to 0.00194 pN/*μ*m s.

## Results/Discussion

Optical tweezers have been used successfully in the studies of RBC elasticity because of the finer-scale in describing whole-cell deformation [2, 64]. Numerical simulations have mimicked this experimental setup by directly applying stretching forces on the opposite sides of a RBC [23, 33, 59]. In a normal RBC, proper transmembrane protein and protein-to-lipid linkages in the membrane could sustain the cell elasticity and mediate the association between the lipid bilayer and the spectrin network even though a relatively large tensile force is imposed on the cell membrane. However, additional vertical linkages between the lipid bilayer and cytoskeleton domains introduced by parasite proteins in *Pf*-RBC will reduce cell viscoelasticity and enhance cell stiffness. On the other hand, defects in the membrane proteins of RBCs in HS and HE will weaken the bilayer-cytoskeleton interactions and facilitate the detachment of the lipid bilayer from the spectrin network. In this study, we have focused on two types of pathological RBCs, *Pf*-RBCs and hereditary diseases with protein defects, and quantitatively investigate their morphological and biomechanical properties during the stretching test by the two-component whole-cell model.

### Deformability of *Pf*-RBCs

**Fig 4:**
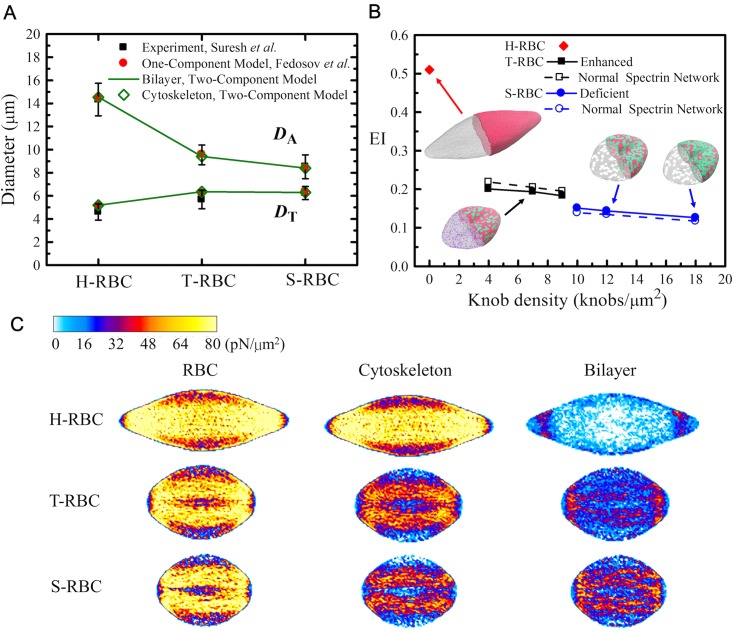
Shape deformation and corresponding stress field of H-RBCs, T-RBCs and S-RBCs. (**A**) The axial (*D*_A_) and transverse (*D*_T_) diameters of H-RBC, T-RBC, and S-RBC at stretching force F_*s*_ = 110 pN. For comparison, the stretching responses in experiments from Ref. [2] and one-component whole-cell model from Ref. [24] are shown. (**B**) Functional dependence of EI values of T-RBCs and S-RBCs on knob density at stretching force F_*s*_ = 110 pN. (**C**) Corresponding stress contours of stretched H-RBC, T-RBC (*ρ*_knob,DPD_≈ 7 knobs/*μ*m^2^), and S-RBC (*ρ*_knob,DPD_≈ 12 knobs/*μ*m^2^).

Here we investigate the elastic properties of the modeled RBC in health but also at different stages of malaria. To probe the RBC mechanical response and the change of its mechanical properties at different malaria stages, we subject the cell to stretching deformation analogously to that in optical tweezer experiments. As shown in Fig 2D, the T-RBC has a lower knob density (*ρ*_knob,DPD_ = 7 knobs/*μ*m^2^) and enhanced spectrin network, whereas the S-RBC bears a higher knob density (*ρ*_knob,DPD_ = 12 knobs/*μ*m^2^) and deficient spectrin network. The total stretching force, F_*s*_, is applied in opposite direction to *ϵN*_*v*_ (*ϵ* = 0.05) vertices of the cell membrane at diametrically opposite directions. First, we examine the response of H-RBCs and *Pf*-RBCs to large deformation in comparison with previous experimental measurements [2] and computational simulations based on the one-component whole-cell model [24]. We analyze the changes in *D*_A_ and *D*_T_. Our simulation results show that both *D*_A_ and *D*_T_ are in agreement with previous experimental and computational results, see Fig 4A. Since the model has separate components for the cytoskeletion and lipid bilayer, we can get the *D*_A_ and *D*_T_ values for each component. The consistency of the *D*_A_ (or *D*_T_) values calculated from the lipid bilayer and the cytoskeleton indicate the strong association between the two components.

To investigate the influence of the knob density on RBC deformability, we change the knob density from 4 to 9 knobs/*μ*m^2^ for T-RBC and 10 to 18 knobs/*μ*m^2^ for S-RBC. For an H-RBC under tensile force F_*s*_ = 110 pN, we obtain that the EI value is about 0.51. For a less deformable *Pf*-RBC, we find a considerable decrease in EI value, *i.e.*, EI ≈ 0.20 for T-RBC at *ρ*_knob,DPD_ ≈ 4 knobs/*μ*m^2^ and EI ≈ 0.15 for S-RBC at *ρ*_knob,DPD_ ≈ 10 knobs/*μ*m^2^. The decrease in EI values from H-RBC to T-RBC then to S-RBC is associated with a reduction of RBC deformability as the progression of the parasite maturation in *Pf*-RBC. With the increase of knob density, a further decrease in EI values for both T-RBC and S-RBC is obtained, see Fig 4B, which indicates a further reduction in cell deformability of the *Pf*-RBC. Our simulation results demonstrate that the knobs, being rigid, contribute to cell membrane stiffness.

In other words, we model the *Pf*-RBC with normal spectrin network by removing the influence of actin remodeling. We find that the EI values increase slightly for T-RBC but decrease slightly for S-RBC (Fig 4B). Nevertheless, in comparison with the change of EI values resulting from the stiffening effects of knobs, the difference of EI values between the modified (enhanced or deficient) and normal spectrin network is relatively small. These simulation results further demonstrate that the presence of the knobs in *Pf*-RBCs is the primary stiffening factor in the loss of cell deformability, which is also consistent with the recent CGMD simulation study by Zhang *et al.* [10]. More importantly, we efficiently scale up the particle-based RBC model from a portion of cell membrane to a whole-cell structure leading to more realistic simulations of RBC dynamics and blood rheology.

Using the virial theorem, we can obtain the average virial stress tensor over a volume Ω,

$$\Pi =\frac{1}{\Omega }\sum_{i\in \Omega }\left[-m_{i}\left(v_{i}-\bar{v}\right)\left(v_{i}-\bar{v}\right)+\frac{1}{2}\sum_{j\in \Omega }r_{ij}\cdot F_{i}\right],$$ (20)

where *m*_*i*_ and **v**_*i*_ are the mass and velocity of particle *i*, respectively, and $\bar{v}$ is the average velocity of particles in the volume Ω. The stress contours of H-RBC, T-RBC, and S-RBC at F_*s*_ = 110 pN are shown in Fig 4C. In general, large stress response is observed around the longitudinal axis of the stretched cell, by contrast, small stress response emerges at the two sides of the transverse axis [65]. For H-RBC, the lipid bilayer has little shear resistance but large bending stiffness and helps to maintain the membrane surface area. The cytoskeleton is primarily responsible for the shear elastic properties of the RBC. Consequently, the principal stress of the stretched RBC is essentially reflected on the cytoskeleton, while only a small stress response is observed at the ends of lipid bilayer where the tensile force is imposed. As shown in Fig 4C, more than 90% of RBC’s total tensile stress comes from the cytoskeleton component, while the rest (less than 10%) from the lipid bilayer component. For T-RBC and S-RBC, the presence of the knobs immersed in the lipid domain stiffens the cell membrane, which is reflected in the stress contour of the lipid bilayer of the stretched RBC, see Fig 4C. The lipid bilayer with stiff knobs contributes ∼ 33% of the total tensile stress of T-RBC membrane. Compared with the results from T-RBC, the knobs with a higher density in S-RBC withstand an elevated stress of the stretched RBC. The stiffened lipid bilayer bears ∼ 45% of the total tensile stress of the S-RBC membrane.

### Simulations of morphology and stress field of RBCs in hereditary spherocytosis and elliptocytosis

The associations between the lipid bilayer and the cytoskeleton mediated by specific molecular interactions are essential for the mechanical stability of RBCs [66]. The primary interaction is the connection between the transmembrane protein band-3 and the spectrin via the ankyrin binding sites. The secondary interaction is supported by another transmembrane protein glycophorin C and the actin junctional complexes. To involve these bilayer-cytoskeleton interactions into the two-component whole-cell model, we have simply considered them together as normal (elastic) and tangential (friction) interactions. Two parameters, the elastic interaction coefficient, *k*_*bs*_ = 46 pN/*μ*m, and the tangential friction coefficient, *f*_*bs*_ = 0.194 pN⋅*μ*m^−1^s^−1^, are accordingly introduced for the normal RBC membrane [45].

We examine cell biomechanics and deformation of H-RBCs subject to stretching tests with the default values of *k*_*bs*_ = 46 pN/*μ*m and *f*_*bs*_ = 0.194 pN⋅*μ*m^−1^s^−1^ of the bilayer-cytoskeleton interactions. First, we probe the stretching deformation of both the lipid bilayer and the cytoskeleton under different stretching force (F_*s*_), see S3 Fig. From this figure, we find that the values of *D*_A_ and *D*_T_ obtained from the two-component whole-cell model are in agreement with those from experimental measurements [2], and the one-component whole-cell model [21]. In addition, we find that there is a small difference in the detachment length, which is defined as the distance along the longitudinal axis from the rightmost part of the lipid bilayer to that of the cytoskeleton. However, the deviations are sufficiently small or purely due to statistical fluctuations under normal stretching forces (F_*s*_ ≤ 200 pN); hence, there is no significant bilayer-cytoskeleton detachment in these cases. A visible detachment of the lipid bilayer from the cytoskeleton appears in extreme cases (for example, F_*s*_ > 300 pN) due to abnormal stretching forces exerted on the cell membrane. Nevertheless, under normal conditions, the large deformability and the strength of the bilayer-cytoskeleton interactions of the H-RBC can be computationally described by the two-component whole-cell model.

**Fig 5:**
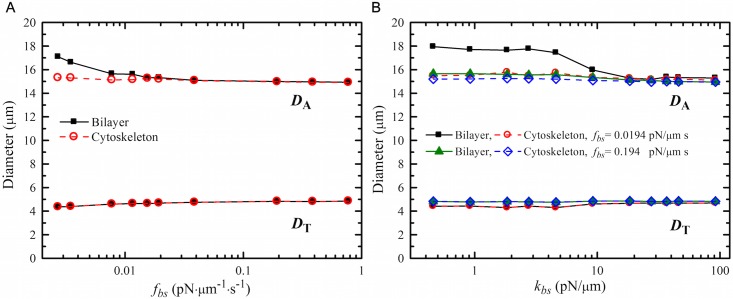
Stretching responses of RBCs at stretching force F_*s*_ = 140 pN as a function of (A) tangential friction coefficient, *f*_*bs*_, and (B) elastic interaction coefficient, *k*_*bs*_. In this figure, *f*_*bs*_ is ranged from 0.00194 to 0.194 pN⋅*μ*m^−1^s^−1^, and *k*_*bs*_ from 0.46 to 46 pN/*μ*m.

**Fig 6:**
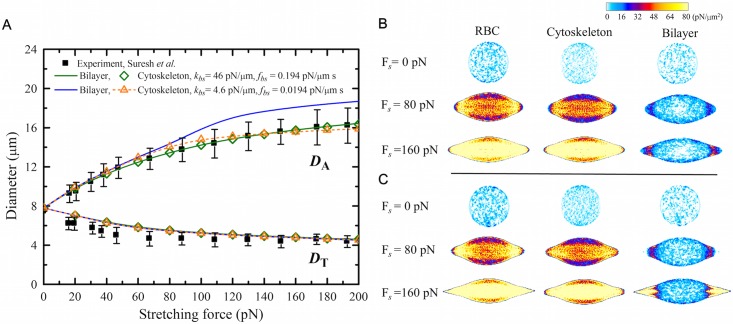
(A) Stretching response and (B-C) corresponding stress field of H-RBCs and defective RBCs under different stretching force. The black squares show experimental results from Ref. [2]. The stress contours of (**B**) H-RBCs and (**C**) defective RBCs at stretching force F_*s*_ = 0, 80, and 160 pN are shown.

To investigate the effect of weakened bilayer-cytoskeleton interactions in defective cell membrane on the deformability of RBCs with stretching test, we present an extensive simulation study on the biomechanical behavior of defective RBCs by varying the values of bilayer-cytoskeleton elastic interaction coefficient, *k*_*bs*_, and tangential friction coefficient, *f*_*bs*_, see Figs 5 and 6. First, we consider the cell membrane with the default value of *k*_*bs*_ but different *f*_*bs*_ (Fig 5A). Specifically, we chose the value F_*s*_ = 140 pN, for which the RBCs undergo large biomechanical deformation without bilayer-cytoskeleton detachment under normal physiological condition. We find that when using the default value of *f*_*bs*_ = 0.194 pN⋅*μ*m^−1^s^−1^ or other values of similar magnitude, there is a strong coupling between the lipid bilayer and the cytoskeleton. This is indicated by the overlapped black solid lines (with black squares) and red dashed lines (with red spheres) in Fig 5A. However, assuming a pathological RBC state where *f*_*bs*_ is decreased by one or two orders of magnitude, an apparent uncoupling between bilayer and cytoskeleton occurs. Compared to the *D*_A_ value of cytoskeleton, the *D*_A_ value of the lipid bilayer is relatively larger when *f*_*bs*_ is smaller than 0.01 pN⋅*μ*m^−1^s^−1^, and the detachment length between the lipid bilayer and the cytoskeleton increases as *f*_*bs*_ reduces. Moreover, the *D*_T_ values obtained from the lipid bilayer and cytoskeleton remain almost the same regardless of the changes in *f*_*bs*_, which can also be observed in Fig 5A. It seems that the *D*_T_ value is insensitive to the variation of *f*_*bs*_. Next, we study the effect of the bilayer-cytoskeleton elastic interaction coefficient as shown in Fig 5B. Similarly, there is no obvious distinction in the deformation of RBC for different *k*_*bs*_ at the default value of *f*_*bs*_ (0.194 pN⋅*μ*m^−1^s^−1^). However, the bilayer-cytoskeleton uncoupling along the stretching axis occurs when we decrease the values of both *f*_*bs*_ and *k*_*bs*_ by one or two orders of magnitude. Consistent with the previous simulations on RBC traversing across microfluidic channels and tank-treading dynamics [45, 46], the strength of the bilayer-cytoskeleton interaction responsible for the association between lipid bilayer and cytoskeleton is again verified by the RBC stretching tests.

Next we compare our simulation results with experimental data for normal and defective RBCs. The results on the functional dependence of the stretching response of normal and defective RBCs on the stretching force, F_*s*_, are shown in Fig 6A. In general, *D*_A_ grows while *D*_T_ decreases with increasing F_*s*_. For H-RBC, no bilayer-cytoskeleton detachment is observed due to the strong bilayer-cytoskeleton interactions. The corresponding stress contours of deformed RBCs at F_*s*_ = 0, 80 and 160 pN are presented in Fig 6B. The tensile stress of H-RBC inherently coming from the cytoskeleton is enhanced as F_*s*_ rises, which is in reasonable agreement with other computational results [65]. On the contrary, the lipid bilayer is observed to separate from the underlying spectrin network when a defective RBC undergoes large deformation due to the weakened bilayer-cytoskeleton interactions (Fig 6A). The stress contours of the defective RBC at F_*s*_ = 160 pN in Fig 6C show significant shear response at two ends of the stretching sites in the lipid domains coinciding with the location for bilayer-cytoskeleton detachment.

**Fig 7:**
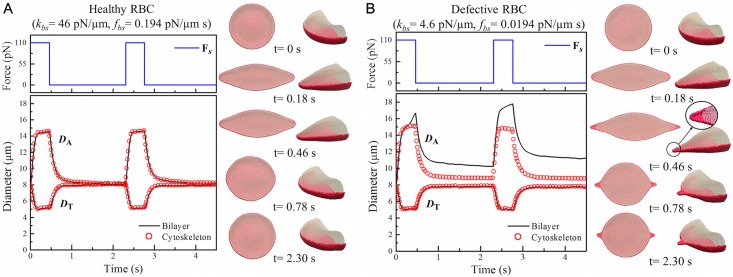
Variation of axial (*D*_A_) and transverse (*D*_T_) diameters of (A) H-RBCs and (B) defective RBCs at stretching force F_*s*_ = 110 pN under stretching-relaxation cycles. Snapshots of RBC shape at time *t* = 0, 0.18, 0.46, 0.78, and 2.30 s are shown.

Upon external tensile forces, a normal RBC undergoes large mechanical deformation, and it restores to its original state when the external tensile force is no longer applied. However, the weakened bilayer-cytoskeleton interactions due to the vertical or lateral defects disturb the biomechanical stability of the RBC membrane. Therefore, when a defective RBC experiences large biomechanical deformation, it may be unable to recover its original shape even though the stretching force causing the shape change is removed. Here, we examine the elastic relaxation of H-RBC and defective RBC when F_*s*_ = 110 pN is turned off. The simulation results are shown in Fig 7 and more details are available in the video clips in the Supporting Information. In Fig 7A, we find that both the lipid bilayer and the cytoskeleton are deformed simultaneously under stretching-relaxation cycles and their *D*_A_ and *D*_T_ values approach the initial values at equilibrium state, which demonstrates that a normal RBC with high elasticity can recover its original shape. The recovery process of the RBC can be characterized by the dynamic recovery expression, R(t), which is described by an exponential decay [67],

$$R\left(t\right)=\frac{\left(\lambda -\lambda_{\infty }\right)\left(\lambda_{0}+\lambda_{\infty }\right)}{\left(\lambda +\lambda_{\infty }\right)\left(\lambda_{0}-\lambda_{\infty }\right)}=\text{exp}\left[\frac{-(t-t_{0})}{t_{c}}\right],$$ (21)

where $\lambda =\frac{D_{A}}{D_{T}}$, *λ*_0_ and *λ*_∞_ correspond to the ratios at release and recovery states; *t*_0_ is the time when F_*s*_ is turned off and *t*_*c*_ is the characteristic time. Using the relaxation results from Fig 7A into R(t), we obtain the best fitting decay as illustrated in S4 Fig and estimate the characteristic time, *t*_*c*_, for both lipid bilayer and cytoskeleton, *i.e.*, *t*_*c*_ ≈ 0.12 s. This value falls within the range of 0.1–0.3 s measured from the shape relaxation of H-RBCs in experiments [65, 67] and also computed in simulations [33].

However, for the defective RBC with weakened bilayer-cytoskeleton interactions, once the detachment of the lipid bilayer from the cytoskeleton occurs, the *D*_A_ values of both lipid bilayer and cytoskeleton are always larger than those at equilibrium state, even for a sufficient relaxation time, which indicates that the defective RBC loses its ability to recover its original shape. We note that not only the lipid bilayer but also the cytoskeleton has a permanent deformation because of the slow response distortion as shown in Fig 7B and the S2 video.

In summary, our simulation results show that the elastic deformations of *Pf*-RBCs match those obtained in optical tweezer experiments for different stages of intraerythrocytic parasite development. We found that the developed knobs on the cell membrane can effectively stiffen the lipid bilayer and the spectrin network, leading to a decrease in RBC deformability. We also investigated the effects of bilayer-cytoskeleton interactions and our simulation results reveal that these interactions play a key role in the determination of cell membrane biomechanical properties. We analyzed the biomechanical response of the RBCs during loading and upon release of the tensile force, in order to mimic the stretching and compression that RBCs experience as they pass through small capillaries in the microcirculation. Our results indicate that H-RBCs with normal bilayer-cytoskeleton interactions recover their original discocyte shape when the stretching force is turned off. However, defective RBCs with weakened bilayer-cytoskeleton interactions lose their ability to recover their original shape due to the irreversible membrane damage. Overall, our findings demonstrate that the *two-step multiscale framework* we have developed, combining coarse-grained molecular dynamics (CGMD) and dissipative particle dynamics (DPD), can be used to predict the altered biomechanical properties of RBCs associated with their pathophysiological states.

## Supporting Information

S1 Fig Shear stress-strain responses of H-RBC and HE-RBC membrane with various cytoskeleton connectivities. (TIF)

S2 Fig Shear stress-strain responses of RBC membrane of T-RBC with *ρ*_knob,CGMD_ = 20 knobs/*μ*m^2^ and enhanced spectrin network, and S-RBC with *ρ*_knob,CGMD_ = 50 knobs/*μ*m^2^ and deficient spectrin network. (TIF)

S3 Fig Stretching responses of H-RBC (*k*_*bs*_ = 46 pN/*μ*m and *f*_*bs*_ = 0.194 pN⋅*μ*m^−1^s^−1^) under different stretching force. Comparisons with the experimental results from Ref. [2], and one-component whole-cell model from Ref. [21].
(TIF)

S4 Fig Dynamic recovery R(t) of lipid bilayer and cytoskeleton of H-RBC after the stretching force, F_*s*_ = 110 pN, is turned off. The characteristic time, *t*_*c*_, estimated from the fitting curves is around 0.12 s. (TIF)

S1 Table The elongation index (EI) of S-RBC with different knob density and spectrin deficiency at F_*s*_ = 110 pN. (TIF)

S1 Video Dynamic stretching–relaxation process of a healthy RBC with normal bilayer-cytoskeleton interaction under the stretching force F_*s*_ = 110 pN. (AVI)

S2 Video Dynamic stretching–relaxation process of a defective RBC with weakened bilayer-cytoskeleton interaction under the stretching force F_*s*_ = 110 pN. (AVI)
