# Supplementary figures and images for: MD/DPD Multiscale Framework for Predicting Morphology and Stresses of Red Blood Cells in Health and Disease
Source: PLoS Comput Biol. 2016 Oct 28;12(10):e1005173. doi: 10.1371/journal.pcbi.1005173 (PMC5085038; doi:10.1371/journal.pcbi.1005173)

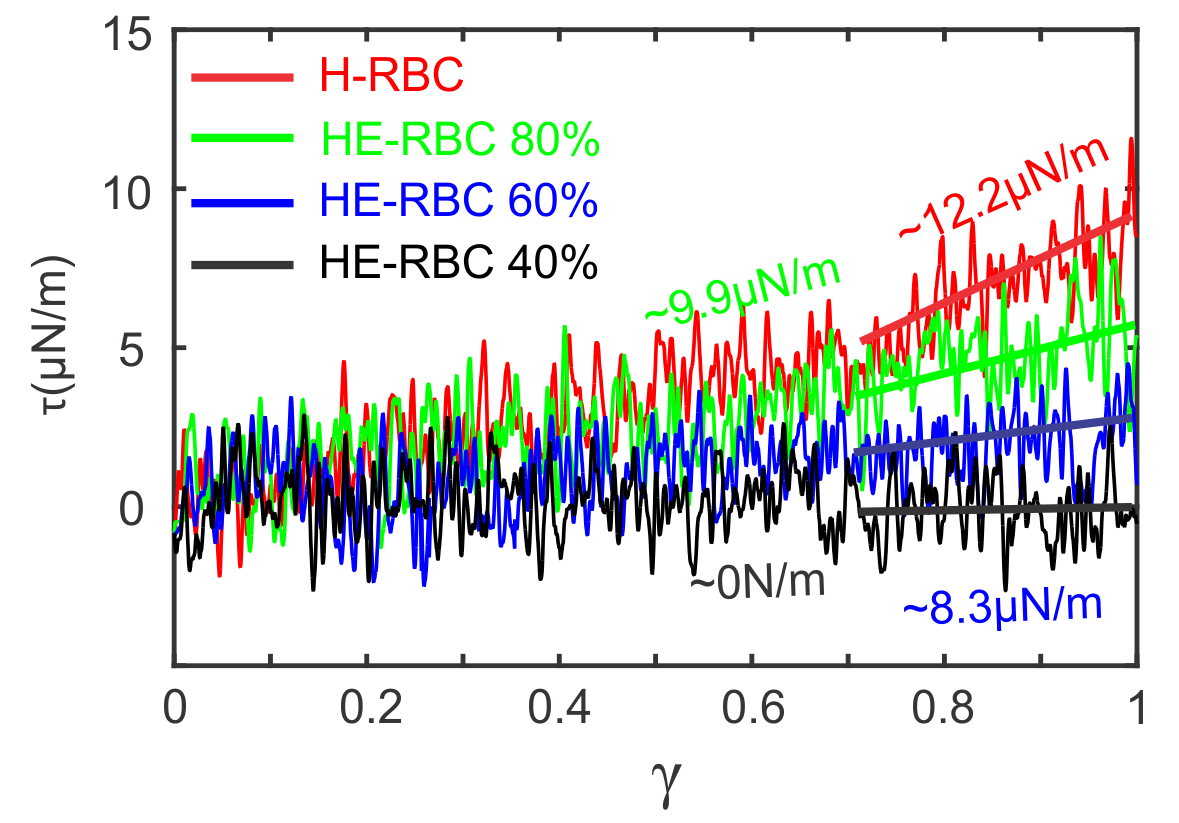

Supplement: S1 Fig — (TIF) [file pcbi.1005173.s001.tif]

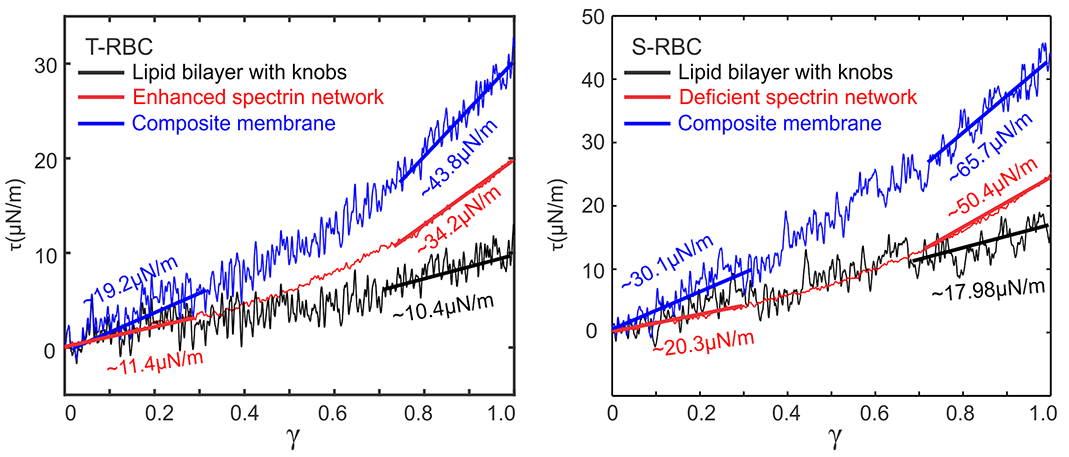

Supplement: S2 Fig — (TIF) [file pcbi.1005173.s002.tif]

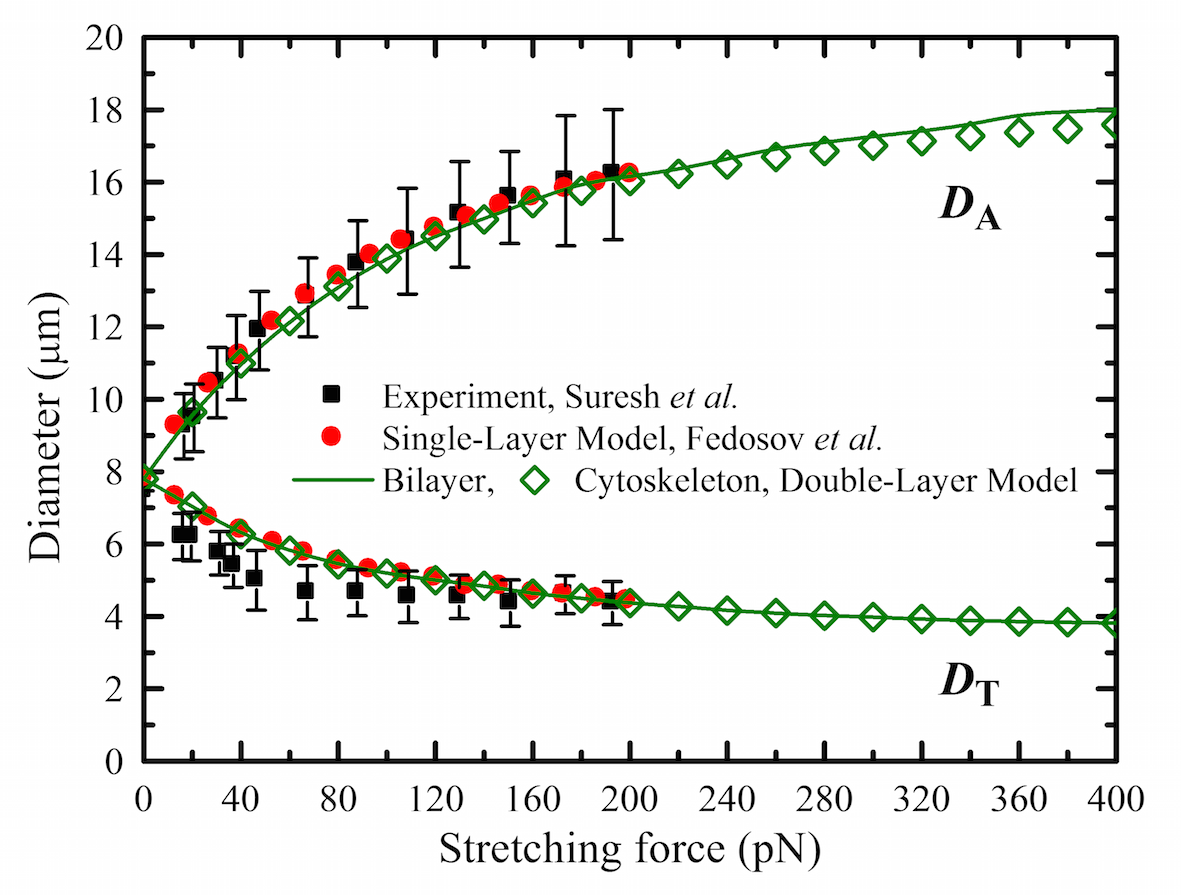

Supplement: S3 Fig — Comparisons with the experimental results from Ref. [2], and one-component whole-cell model from Ref. [21]. (TIF) [file pcbi.1005173.s003.tif]

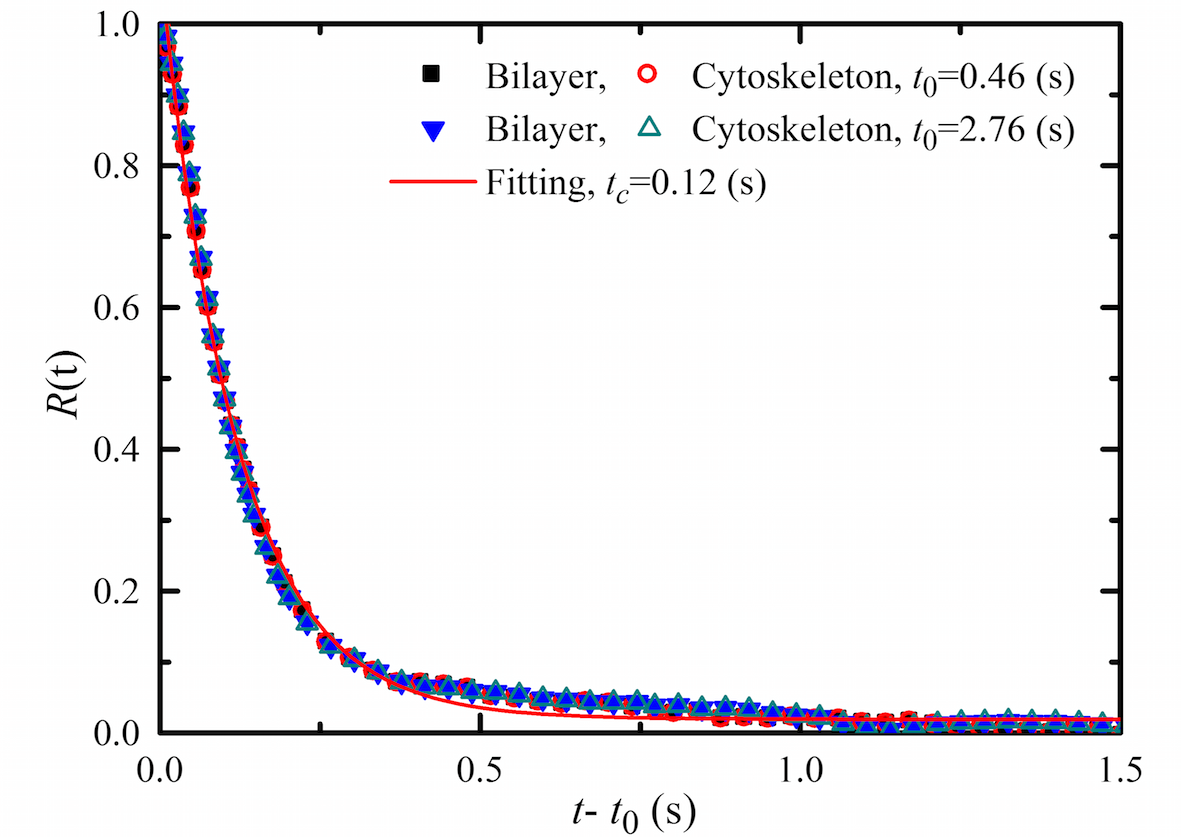

Supplement: S4 Fig — (TIF) [file pcbi.1005173.s004.tif]

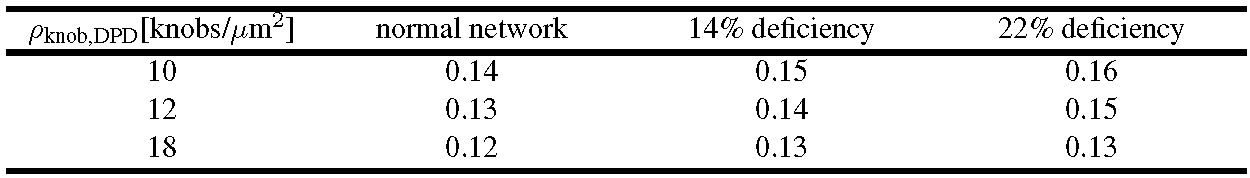

Supplement: S1 Table — (TIF) [file pcbi.1005173.s005.tif]
